# Dengue Virus and Influenza A Virus Co-Infection in Pregnancy: A Case Report

**DOI:** 10.3390/tropicalmed4020084

**Published:** 2019-05-21

**Authors:** Mónica Guzmán-Rodríguez, Héctor F. Acosta-Ñañez, Julio César Mantilla, Anilza Bonelo

**Affiliations:** 1 Magister in Biomedical Sciences, Universidad del Valle, Cali 760001, Colombia; 2 Department of Pathology, Universidad del Valle, Cali 760001, Colombia; hefacosta@hotmail.es; 3 Department of Pathology, Universidad Industrial de Santander, Bucaramanga 680001, Colombia; jcmlaboratorio@gmail.com; 4 Emergent Viruses and Disease - VIREM, Universidad del Valle, Cali 760001, Colombia; anilza.bonelo@correounivalle.edu.co

**Keywords:** dengue, Influenza, co-infection, fatal case

## Abstract

Dengue is still an important cause of disease and mortality in tropical countries, as is influenza A virus, which is also a cause of epidemics all over the globe. In this article, we present the case of a 31-year-old woman who was in her second trimester of pregnancy and presented with severe dengue with hematological and neurological complications, and premature labor. She was misdiagnosed with bacterial infection and received antibiotic treatment with no improvement of the clinical manifestations and previous to death, she was diagnosed with dengue infection. She died from cardiorespiratory arrest. In the postmortem evaluation, influenza A co-infection was confirmed and characterization of the tissue damage and immune response in lung, liver, kidney, heart, spleen, and brain was determined, finding a severe inflammatory response in lung with T cells and macrophages infiltrating the tissue. This case report highlights the risks of accepting a single diagnosis, especially in endemic countries to multiple tropical diseases, which can lead to delay in appropriate treatment that could reduce morbidity and mortality.

## 1. Introduction

Dengue is the most prevalent arthropod-borne viral disease in the world. It is estimated that dengue causes around 390 million infections every year and more than 2.5 billion people live in areas at risk [1]. Colombia is an endemic country for dengue, with a permanent circulation of the virus through the year and peaks of incidence in the rainy seasons, where dengue has an incidence of 100 cases per 100,000 habitants and caused approximately 17 deaths in the last year, with a lethality rate of 5.9% [2].

Dengue is caused by any of its four serotypes (DENV-1, -2, -3, -4), and is transmitted by *Aedes* mosquitoes. Usually, it causes a mild self-limited febrile illness but in less than 12% of cases, severe disease can occur, characterized by vascular leakage, severe bleeding, and multiorgan involvement, that can induce shock syndrome, leading 1 to 10% of cases to death [3]. Severe dengue has been associated with virus factors such as virulence; immunological mechanisms such as antibody-dependent enhancement (ADE) which involves the opsonization of DENV particles that are bound in immune complexes with non- neutralizing antibodies, leading to higher levels of viral replication and increase in infection rate in crystallizable fragment (Fc) receptor (FcR)-bearing cells, such as DCs and monocytes. This phenomena has been observed in multiple animal models and in vitro assays, and has been associated with severe dengue in secondary infections; and finally with host factors such as age and comorbidities [1].

Influenza is an important cause of mortality, contributing to one of the top five causes of death in the world [2]. It is estimated that influenza causes 90 million infections in children aged 0–4 years and 27,800 deaths [3]. In Colombia, influenza A virus (INFA) circulates throughout the year, increasing its incidence during the rainy seasons. The most prevalent strain is A (H2N3) followed by A (H1N1), with a mortality rate of 10.7 per 100,000 children younger than 5 years old, and less than 6 per 100,000 adults younger than 65 years old [4]. Influenza causes a respiratory syndrome characterized mainly by fever, cough, myalgia, and headache, but in some cases, depending on the strain and patient characteristics, it can result in lung complications, such as pneumonia, or even extra-pulmonary symptoms, such as heart failure, acute kidney injury or encephalitis, leading to multiorgan failure and eventually to death [5]

Co-infection of DENV with INFA is thought to increase disease severity. In Colombia, the fact that DENV and INFA circulate all year and that both cause epidemics during the rainy seasons, the chance of presenting with both infections is likely, presenting a diagnostic challenge, given that both infections can cause similar symptoms [5]. There have been several reports of severe cases of co-infection but so far, this has not been reported during pregnancy [6,7,8]. 

In this article, we present the case of a pregnant woman who manifested with severe disease, causing both her own death and the death of her newborns, highlighting the importance of adequate awareness of clinicians to detect possible co-infections and the need for larger studies to evaluate if INFA infection is present in fatal or severe dengue cases that might explain the severity in some of those patients.

## 2. Case Presentation

A 31-year-old woman, previously healthy, with pregnancy at 26 weeks, was admitted to a primary health center facility in Santander with a 2-day history of fever, headache and myalgia. The initial physical exam showed blood pressure of 146/92 mmHg without any other abnormality. She had no fever, high pulse rate or petechiae, or any bleeding signs. Laboratory evaluation revealed a total leukocyte count of 7280 cells/mm^3^, hemoglobin of 11 g/dL and platelet count of 61,000 cells/mm^3^. Additionally, proteinuria of 100 mg/dL was detected. The patient was diagnosed with preeclampsia and was transferred to the University Hospital of Santander, a tertiary level hospital, for further evaluation and treatment. 

At admission, biochemical analysis was performed, finding that her aspartate aminotransferase (AST) and alanine aminotransferase (ALT) levels, renal function tests and electrolytes were normal. Treatment with nifedipine and magnesium sulfate was started. At admission and during the follow-up, blood pressure measures were within the normal range, but treatment was administered given the single high blood pressure measure presented in the first clinical evaluation. Also, an ultrasound with fetal Doppler was obtained, showing a fetal heart rate between 110 and 150 beats/min, estimated weight of 862 g (percentile 0.17), amniotic fluid index (AFI) of 16.04 (normal), fetal hemodynamic changes with absence of diastolic flow in the umbilical artery and redistribution phenomenon, brain/placenta pulsatility index (PI) relation in 2.5 percentile, and an average PI of 1.8. Severe intrauterine growth restriction was diagnosed, and lung maturation with steroids was immediately initiated.

On day 2 of hospitalization, the result of the urine culture was received, showing >100,000 CFU of *Enterococcus spp.* sensitive to Ceftriaxone and because the patient persisted with fever, antibiotic treatment was started. Additionally, she started to present respiratory distress and desaturation, with diminution in basal ventilation of the lungs. The new blood evaluation showed a decrease in total leukocyte count to 3660 cells/mm^3^ and in the platelet count to 47,000 cells/mm^3^. Because Santander is an endemic area for dengue, the new suspicion was dengue infection. IgM specific for dengue was requested. 

On day 3, the patient started labor spontaneously. A male was born, with a weight of 730 g and length of 34 cm. He was transferred to the neonatal intensive care unit where he died with cerebral hemorrhage and respiratory distress. Before child birth, two blood units and six platelet units were transfused. After the birth, five new blood units were transfused to the patient. The control platelet count after the transfusions was 37,000 cells/mm^3^. 

In the immediate post-partum, hepatomegaly was detected, with superficial breathing and basal stertorous. The patient started to present visual and auditive hallucinations with agitation periods. Chest x-ray showed bilateral pleural effusion. Additional treatment with clarithromycin was initiated, with a presumptive diagnosis of complicated pneumonia. Initiation of mechanical ventilation and vasoactive with inotropic support were required. The dengue IgM result was positive on day 4. On day 5, the patient presented respiratory dysfunction and failure to respond to inotropic treatment, ultrasonography and computed axial tomography discarded the presence of pulmonary embolism. Finally, the patient died from cardiorespiratory arrest. Table 1 shows the follow-up of the laboratory results available in the clinical records.

The autopsy revealed pleural effusion, pericardial effusion and hemorrhage at the base of the skull and in the right lobe of the liver, with changes associated with microangiopathic thrombosis, which is commonly associated with dengue virus. The Colombian National Institute of Health received samples of the tissues for analysis, finding Dengue IgG negative, IgM positive and a positive RT-PCR for DENV-2, confirming the case as a fatal dengue case with a primary infection. 

Paraffin-embedded formalin-fixed tissues from the lung, liver, heart, brain, kidney and spleen of the patient were obtained. Initially, a histological evaluation with hematoxylin/eosin staining was performed, followed by immunopathological evaluation with immunohistochemistry using different cellular markers in order to identify the cellular infiltration and its relationship with tissue damage. Briefly, the tissues underwent deparaffinization with xylene, rehydration in an alcohol battery, antigen retrieval with citrate buffer pH 6.0, blocking of endogenous peroxidases with hydrogen peroxide at 3%, blocking with normal goat serum 3%, incubation with primary antibody, posterior addition of secondary antibody from goat, incubation with Avidine/Biotin-ABC Kit (VECTOR/PK-6101 Peroxidase) and revealing of the reaction with DAB Kit (VECTOR/SK-4100).

**Figure 1:**
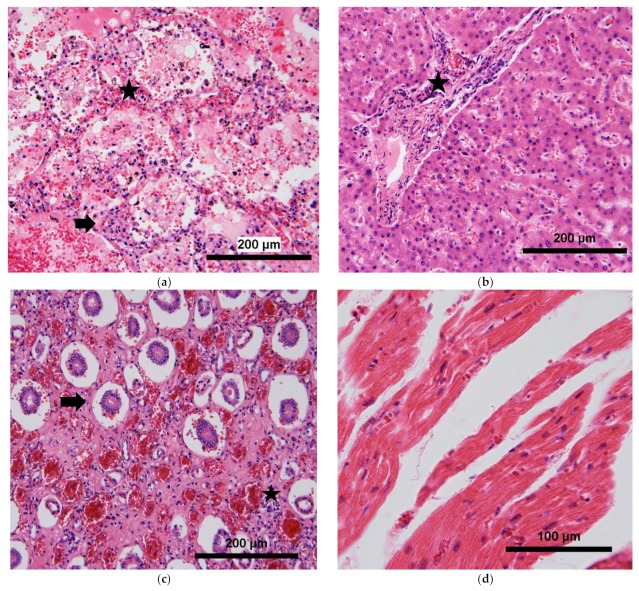
Representative pictures of the main findings in the histological evaluation of the tissues. (**a**) Lung tissue with alveolar septa damage and hemorrhage (star), associated with scarce inflammatory lymphocytic infiltrate in septa (arrow); (**b**) Liver tissue with moderate inflammatory infiltrate in portal triad (star) and sinusoidal edema; (**c**) Kidney tissue with vascular congestion (arrow), atrophy and tubular necrosis, associated with moderate inflammatory infiltrate (star) and signs of stromal fibrosis; (**d**) Heart tissue with interstitial edema and vascular congestion.

The primary antibodies used in immunohistochemistry were: rabbit polyclonal anti-CD3 at a dilution of 1:200 (DAKO A045201), mouse monoclonal anti-CD4 at a dilution of 1:100 (DAKO M731001), mouse monoclonal anti-CD8 at a dilution of 1:100 (DAKO M710301), mouse monoclonal anti-CD68 at a dilution of 1:100 (DAKO M087601) and mouse monoclonal anti-CD20 at a dilution of 1:100 (DAKO M075501). The secondary antibodies used were biotinylated goat anti-rabbit at 1:200 and goat anti-mouse at 1:200. These stained tissues were evaluated by two different pathologists, with a concordance index of 0.95. The main findings in these tissues are described in Table 2 and shown in Figure 1.

**Figure 2:**
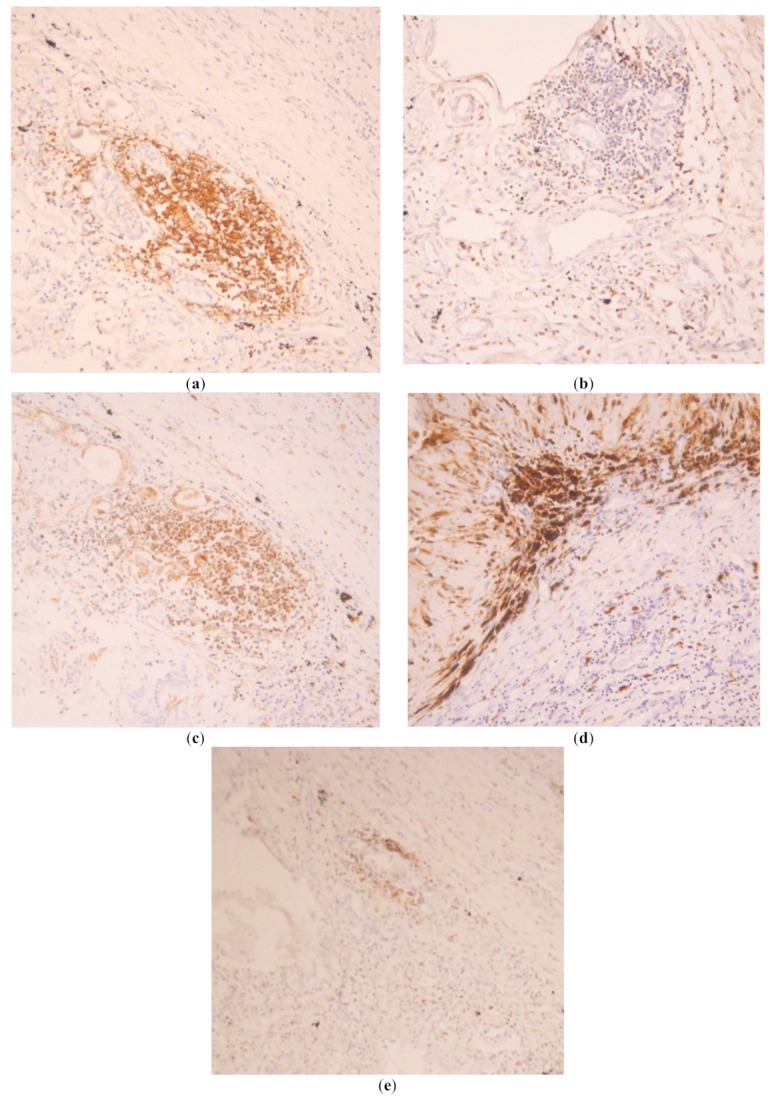
Representative pictures of the immunohistochemistry of the lung. Positive cells are colored brown. (**a**) Abundant T cells CD3^+^ in perivascular location; (**b**) Scarce CD4^+^ T cells; (**c**) Abundant HLA-DR^+^ T cells. Most of the T cells were activated through the expression of this cell marker; (**d**) abundant CD68+ cells (macrophages) affecting alveoli and interstice; (**e**) Scarce CD20+ cells (B cells), located in the perivascular zone.

The main organ affected was the lung, with the presence of edema, hemorrhage, alveolar damage and vascular congestion (Figure 1a). The main cell types infiltrating the tissue were T cells in septa and alveoli, predominantly CD8^+^ T cells, with an inversion of the ratio CD4^+^/CD8^+^ (Figure 2a,b). These T cells were found to express high levels of HLA-DR, a marker of T cell activation (Figure 2c). Macrophages were also found abundantly in the lung, mainly in alveoli (Figure 2d). B cells were present to a lesser extent, with multiples focus in septa all over the tissue (Figure 2e). Neutrophils were not evaluated with specific staining but, in the histological evaluation, were found infiltrating septa in moderate amounts.

To discard another co-infection, DNA and RNA from the tissues were extracted with Kit FFPE DNA/RNA QIAGEN^®^ to perform conventional PCR for the detection of *Lepstospira spp*. [9] and *Plasmodium spp*. [10]. Additionally, real-time RT-PCR (qRT-PCR) was made for the detection of Zika virus [11], Chikungunya virus [12], influenza B virus and influenza A virus [13]. All the tests were made in duplicate, with a negative control from the extraction and a negative control from the RT-PCR process. Primers used for these experiments were designed and standardized elsewhere [9,10,11,12,13]. Influenza A infection was confirmed in the lung tissue, in two different experiments, with an average Cycle Threshold CT of 35 (cut-off point of 38.5), and the negative controls showed no amplification curve.

## 3. Discussion

During pregnancy, changes in mechanical and pathophysiological aspects in several organs occur, such as a decrease in respiratory volume and relative immune suppression, which increase the susceptibility to infections and the risk of complications [14].

The effects of dengue infection during pregnancy on the mother and the fetus are not clear. It is thought to be associated with increased risk of complications. In one retrospective study, 20 pregnant women with dengue fever were evaluated, finding that low platelets and high liver enzymes, which are also diagnostic criteria for HELLP (Hemolysis, Elevated Liver enzymes, Low Platelets) Syndrome, were associated with higher risk of postpartum hemorrhage [15]. Another prospective cohort study in French Guiana, which included 73 exposed pregnant women to dengue virus infection, also found high rates of hemorrhage during labor or in the immediate postpartum, with 8.6 times higher risk than in the group of non-infected women [16]. Adverse fetal outcomes are not consistently associated with dengue infection. But in a systematic review, associations between dengue infection with miscarriage, preterm birth and low birthweight were established [17]. Influenza A infection has been associated with severe outcomes in pregnancy, evidenced especially during influenza pandemics, when mortality can rise up to 50% in pregnant women, and it is also associated with complications in the fetus when presented during the pregnancy, especially in the first trimester [18].

Co-infection of DENV with INFA is thought to increase disease severity. There have been several reports of severe cases of co-infection. In El Salvador, during influenza season, 123 patients with suspected dengue were evaluated, finding that 19% tested positive for influenza and three cases were confirmed as a co-infection DENV-INFA. However, in this study, the severity of the disease was not evaluated [6].

In animal models, it was determined that infection with INFA followed by DENV infection increases DENV titers in lung, liver and spleen, increases the risk of severe pneumonia and impairs the recruitment of monocytes to the lung, augmenting neutrophil infiltration to the lung, finally leading to a higher mortality rate [7]. But there are no studies of the pathology of DENV-INFA co-infection in humans.

In this case, we presented a woman in her second trimester of pregnancy, with no record of previous disease, who presented with unspecific symptoms, such as fever, myalgia, headache and cough, which can easily be confounded with several tropical infectious diseases present in Colombia and even with preeclampsia, autoimmune and neoplastic diseases. Diagnosis of preeclampsia was determined and the patient received adequate treatment for this pathology, with control of blood pressure measures. However, it is important to highlight that preeclampsia has also been associated with complications such as those presented by the patient (hemorrhage, respiratory distress and disseminated intravascular coagulation) and could explain, in part, the severity of the disease.

Interestingly, dengue fatality is usually associated with secondary infections. However, given the serological status of the patient, this was a primary dengue infection and mechanisms of severity could be related to influenza A co-infection and the association to preeclampsia.

We found that, in multiple organs, there were several pathological changes, especially in lung and liver, with abundant infiltration of mononuclear cells, particularly T cells and macrophages, associated with severe inflammatory changes such as edema, hemorrhage and vascular congestion, which have been associated with immunopathological mechanisms of damage in dengue and in influenza infections, and can be related with the poor outcome of the patient.

The diagnosis of the co-infection could have been decisive in the treatment of the patient, because, although there is not a specific treatment for dengue, for influenza infection, antiviral drugs such as oseltamivir have proven to be efficient in reducing complications and mortality. Also, prenatal exposure to oseltamivir has not been associated with increased risk of congenital malformation, poor fetal growth, fetal death or preterm birth [19,20], so the treatment of pregnant women not only reduces complications and mortality associated with the infection but has no pathological effect on the child.

Finally, this case report highlights the risk of accepting a single diagnosis, and the need for larger studies to evaluate if influenza co-infection in dengue cases may explain the severity of some of them, because, if there is an epidemiological significance (high co-infection rate among severe cases of dengue), it could have implications in the selection of an effective treatment and reduction of complications and mortality.

## Figures and Tables

**Table 1:** The laboratory results.

Parameter	Day 1	Day 2	Day 3	Day 5	Day 6
**WBC (cells/mm^3^)**	7280	8210	-	8600	4170
**Hemoglobin (mg/dL)**	11.1	12.5	-	9	12.1
**Hematocrit (%)**	32.6	35	-	30	36
**Platelets (cells/mm^3^)**	61,000	73,800	37,000	53,000	43,000
**Reactive C Protein (mg/dL)**	-	-	-	385	284
**Aspartate aminotransferase (AST) (mg/dL)**	-	14.6	-	117	67
**Alanine aminotransferase (ALT) (mg/dL)**	-	8.6	-	40	20
**Creatinine (mg/dL)**	-	-	-	0.75	0.81
**Na+ (mg/dL)**	-	-	-	145	155
**K (mg/dL)**	-	-	-	5.8	6.12
**Cl (mg/dL)**	-	-	-	100	116
**Dengue IgM**	-	-	-	Positive	-
**Proteinuria (mg/dL)**	100	300	-	-	25
**HIV tests (Western Blot)**	-	-	-	-	Negative

**Table 2:** Main findings in the histological analysis of the tissues.

	Lung	Liver	Kidney	Brain	Heart	Spleen
**Morphological findings**	Edema Hemorrhage Alveolar damage Vascular congestion	Steatosis Mild fibrosis	Vascular congestion Fibrosis	Edema Hemorrhage Vascular congestion	Vascular congestion Negative for cellular infiltration	Vascular congestion
**Neutrophils**	Moderate	Negative	Negative	Negative	NA	NA
**Macrophages**	Abundant Alveoli	Moderate Sinusoids	Scarce	Negative	NA	NA
**T cells**	Abundant Septa	Scarce Portal	Abundant	Scarce	NA	NA
**B cells**	Abundant Multifocal Septa	10% of lymphocytes Portal	Negative	Negative	NA	NA
**NK cells**	Negative	Mild Portal	Negative	Negative	NA	NA
